# Deep Reinforcement Learning-Based Path-Following Control for Underactuated Autonomous Underwater Vehicles

**DOI:** 10.3390/s26144548

**Published:** 2026-07-17

**Authors:** Xin Pan, Lin Huang, Liangjin Li, Song Wang

**Affiliations:** College of Electrical Engineering, Naval University of Engineering, Wuhan 430033, China; 1920191171@nue.edu.cn (X.P.);

**Keywords:** deep reinforcement learning, Unmanned Underwater Vehicle, path following, underactuated control

## Abstract

Autonomous Underwater Vehicles (AUVs) face significant challenges in path-following control due to strong environmental disturbances and model uncertainties. To address these issues, this paper proposes a model-free deep reinforcement learning framework, named ILLT (Improved LOS-LSTM-TD3), which integrates an integral line-of-sight (LOS) guidance law with the twin delayed deep deterministic policy gradient (TD3) algorithm. The framework treats the LOS look-ahead distance as a learnable optimization variable and incorporates an LSTM network to capture temporal motion dependencies. A progressive unfreezing transfer learning strategy, combined with attention-based feature–current fusion, is designed to enhance domain adaptation under varying ocean currents. Simulation results demonstrate that ILLT reduces the average cross-track error by 48.5% compared to the baseline ILT algorithm and by 66.4% compared to traditional PID control, while achieving significantly faster convergence in target domains. Physical experiments in tank and lake environments further validate the algorithm’s feasibility and robustness, with tracking errors approaching simulation results under moderate current conditions. These findings confirm the effectiveness of the proposed framework for underactuated AUV path-following tasks.

## 1. Introduction

Autonomous Underwater Vehicles (AUVs), as a key subset of Unmanned Underwater Vehicles (UUVs), are important components of modern marine equipment systems, featuring small targets, strong stealth capabilities, and high maneuverability. They have broad application prospects in mine countermeasures, anti-submarine warfare, underwater intelligence gathering, and target reconnaissance [1]. Path-following technology refers to the process in which a vehicle stably tracks a predefined geometric path by computing position deviations in real time and adjusting control inputs (e.g., steering angles and speeds). This technology is crucial in many tasks and is one of the key technologies for precision docking and ocean search [2]. Since the underactuated systems commonly used in AUVs represent a classic challenge in control theory, and considering the complexity and strong nonlinearity of AUV underwater motion, the design of AUV path-following control becomes even more challenging.

Research on path-following technology covers a variety of methods ranging from classical control strategies to artificial intelligence techniques. Among classical control methods, the line-of-sight (LOS) method is frequently adopted as the guidance law for path-following problems. It relies on a virtual target point to guide the path, and its core is to compute the desired heading angle, then combine it with control algorithms to achieve stable path following [3]. Since traditional LOS methods often fail to achieve satisfactory path-following performance, an important research direction is to improve the LOS algorithm. Among these, integral line-of-sight (ILOS) guidance adds an integral term to the traditional LOS guidance law to reduce unknown effects such as ocean currents and eliminate steady-state tracking errors [4,5,6]. Adaptive line-of-sight (ALOS) dynamically adjusts the look-ahead distance in real time based on the current system state (e.g., tracking error, path curvature, speed), achieving regulation of path following [3,7]. Extended state observer-based line-of-sight (ELOS) designs a separate state observer to estimate ocean current disturbances and compensate for them [8,9].

In studies combining LOS guidance laws with control algorithms, Ref. [10] combined a fixed-time guidance strategy with preset performance control and designed an optimal feedback controller, whose solution and optimization were realized through a single critic neural network (SCNN). Ref. [11] addressed the energy consumption problem of AUVs by combining path curvature adaptation and dynamic target optimization, establishing a multi-objective optimization framework to redesign the LOS reference point. Simulations showed that energy consumption was effectively reduced while maintaining tracking accuracy. Ref. [12] constructed a motion controller based on LOS and backstepping to handle unknown currents and external disturbances, and designed a current observer for online estimation of current velocity; simulation results verified the effectiveness and robustness of the control strategy. Ref. [13] proposed a novel indirect adaptive disturbance observer (IADO)-based LOS guidance law, which estimates and compensates for uncertainties caused by ocean currents along curved paths, and an adaptive look-ahead distance is proposed for better-following curved paths with sharp turnings; simulation studies demonstrated good tracking accuracy and anti-disturbance capability. Compliance route planning for unmanned surface vehicles has also received extensive attention; Ref. [14] designed a local path re-planning strategy that satisfies collision avoidance rules at sea, enabling real-time update of safe routes under dynamic obstacle disturbances.

Control of underactuated AUV systems faces strong nonlinearity, parameter uncertainties (e.g., hydrodynamic coefficient deviations), and unknown underwater forces (e.g., second-order wave forces). Traditional nonlinear control methods rely heavily on accurate mathematical models of the controlled object, and thus often fail to achieve good performance in underactuated AUV path-following problems. Reinforcement learning allows an agent to learn optimal policies through trial-and-error interactions with the environment. Model-free reinforcement learning avoids reliance on accurate mathematical models; it does not learn or use any environmental model, and interaction data are used to directly update value functions or policies [15]. In recent years, with the development of artificial intelligence, deep reinforcement learning (DRL), which combines the perception capability of deep learning with the decision-making capability of reinforcement learning, has been applied in AUV control. To address the insufficient generalization ability of AUV obstacle avoidance decision-making in dynamic underwater environments, Cao et al. [16] proposed a fuzzy reinforcement learning-based obstacle avoidance strategy, using a multi-step temporal-difference algorithm for model-free online learning and fuzzy theory to handle state space continuity, significantly improving motion smoothness and adaptability. To quantify collision risks during navigation, Ref. [17] built a risk inference system compliant with maritime collision regulations, achieving active obstacle avoidance decisions based on real-time encounter assessment. In AUV path planning and target tracking, Xi et al. [18] innovatively combined point-cloud observations with reinforcement learning, constructing a leader–follower dual-AUV cooperative system and employing the soft Actor–Critic algorithm for swarm intelligence control. To address communication challenges in cooperative multi-AUV data collection in complex underwater environments, Zhang et al. [19] developed the SLDN method that integrates staged deep reinforcement learning with distributed negotiation, significantly reducing communication overhead while maintaining cooperative efficiency and adaptability to time-varying marine environments. Chen et al. [20] focused on multi-agent cooperative control, proposing a global feature-enhanced reinforcement learning framework with an augmented Critic network to overcome bottlenecks such as global–local information dimension imbalance and slow convergence in the centralized training with decentralized execution framework; this study accelerates convergence through a global state-policy feature alignment mechanism that fuses global and individual AUV information. Zhang et al. [21] focused on improving the robustness of AUV three-dimensional autonomous docking control under currents and wave disturbances, developing a model-free docking controller based on the Proximal Policy Optimization (PPO) algorithm, and introducing two innovative mechanisms—adaptive rollback clipping and self-generated demonstration replay—to significantly enhance the learning efficiency and stability of the PPO algorithm, which is expected to effectively extend AUV operational endurance. Research on path optimization for marine unmanned vehicles has formed a complete system; Namgung et al. reviewed the development of path planning and obstacle avoidance technologies for surface ships under maritime navigation regulations, systematically summarized the applicable scenarios of various intelligent planning algorithms, and proposed a large-model-assisted navigation framework, which can provide references for multi-constrained path-following design for underwater AUVs [22].

Reinforcement learning also offers new ideas and methods for AUV path-following problems. To address the data efficiency bottleneck of AUV path following under unknown ocean currents, Li et al. [23] developed the CMQL algorithm based on offline Q-learning, enabling the controller to achieve zero-shot transfer without online fine-tuning, accurately predicting motion states within the data distribution, thus providing a practical solution for AUV control under limited data scenarios. To tackle the challenge of 3D path-following control under sparse sensor attacks and measurement disturbances, Qiu et al. [24] combined a continuous-time unscented Kalman filter algorithm with a reinforcement learning framework, effectively reducing the impact of sparse sensor data and measurement disturbances. Ma et al. [25] proposed a neural network model-based reinforcement learning control framework; the Actor–Model–Critic (AMC) architecture learns system state transition characteristics through neural network models, effectively capturing the spatiotemporal dynamics of AUV–environment interactions. Zhang et al. [26] proposed and implemented an MPC-based model-based reinforcement learning (MB-MPC) approach, using MPC planning to overcome current disturbances, effectively improving the sampling efficiency and training speed of AUV path following.

Inspired by the above literature, a novel model-free reinforcement learning framework, the ILLT algorithm, is proposed to address the path-following problem of underactuated AUVs under currents and other unknown disturbances, with full consideration of the algorithm’s transferability across different environments. The superiority of the algorithm is verified in both co-simulation environments and physical experiments. The main contributions of this paper are as follows:

(1) Combining integral LOS with the twin delayed deep deterministic policy gradient (TD3) algorithm: The look-ahead distance in LOS is treated as an optimization variable and fed into the neural network for training. An LSTM network is introduced in the input layer to estimate the trend of the predefined path.

(2) Integrating transfer learning strategy into the neural network: Considering the influence of different currents, the input layer of the neural network is divided into a feature extractor and a current encoder, which are concatenated through an attention network. The feature extractor is updated in a progressive unfreezing manner.

(3) Multi-loss function design: To enhance generalization ability, consistency loss and regularization loss are added to the original Critic and Actor structures of the TD3 algorithm, and the training process is redefined to accelerate the algorithm’s training speed from the source domain to the target domain.

The remainder of this paper is organized as follows. In Section 2, the AUV motion model and the horizontal-plane path-following model are established. In Section 3, the path-following control algorithm based on integral LOS and the improved TD3 algorithm is proposed. In Section 4, simulation experiments under different environments are conducted to compare the performance of different algorithms. In Section 5, physical experiments verify the feasibility of the proposed algorithm.

## 2. Preliminaries

### 2.1. AUV Motion Model Establishment

**Figure 1:**
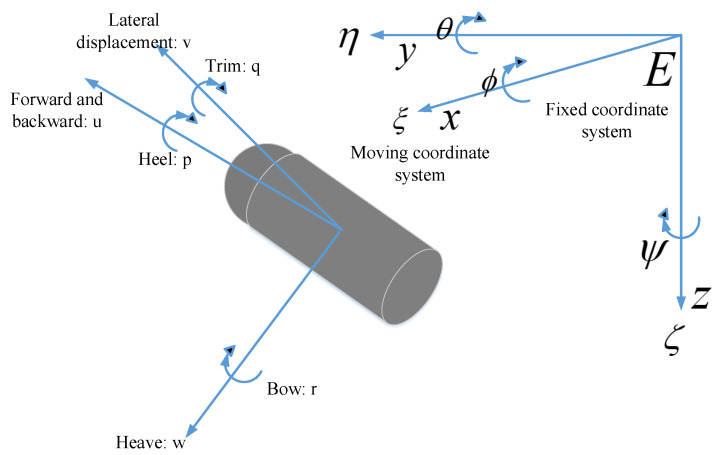
Earth-fixed and body-fixed coordinate systems for the AUV.

To establish the motion model of an underwater vehicle, it is necessary to select appropriate coordinate systems. A body-fixed coordinate system O−xyz and an earth-fixed coordinate system E−ξηζ are introduced to represent the motion state of the AUV [1,7], as illustrated in Figure 1.

The AUV’s six-degree-of-freedom motion in space is generally defined in the earth-fixed coordinate system by the pose vector $\vec{\eta }=[x,y,z,\phi ,\theta ,\psi {]}^{T}$, representing three-dimensional position and Euler angles. The absolute velocity vector of the AUV is generally defined in the body-fixed coordinate system as $\vec{v}=[u,v,w,p,q,r{]}^{T}$, representing linear and angular velocities in three dimensions. Correspondingly, the velocity vectors of the current in the earth-fixed and body-fixed coordinate systems are defined as $V_{c}={\left[v_{c}^{x},v_{c}^{y},v_{c}^{z},0,0,0\right]}^{T}$ and ${\vec{v}}_{c}={\left[u_{c},v_{c},w_{c},0,0,0\right]}^{T}$, respectively. The AUV’s velocity relative to the current can be expressed as: ${\vec{v}}_{r}=\vec{v}-{\vec{v}}_{c}={\left[u_{r},v_{r},w_{r},p,q,r\right]}^{T}$.

The kinematic equations describe the geometric relationship between the AUV’s pose changes and its velocity, expressed as:

(1) $$\dot{\eta }=J(\eta )\nu_{r}+V_{c}$$

where J(η) is the Euler angle-based coordinate transformation matrix, which performs the transformation from the body-fixed coordinate system to the earth-fixed coordinate system.

Based on the fundamental principles of rigid body dynamics and fluid mechanics, the six-degree-of-freedom nonlinear dynamic equations of the AUV can be formulated as [3]:

(2) $$M{\dot{\nu }}_{r}=\tau +\tau_{d}-C(\nu_{r})\nu_{r}-D(\nu_{r})\nu_{r}-g(\eta )$$

where M is the inertia matrix (including added mass); $C\left(\nu_{r}\right)$ is the Coriolis and centripetal matrix; $D\left(\nu_{r}\right)$ is the damping matrix; g(η) is the restoring force vector; and the parameters within these matrices are hydrodynamic coefficients. τ represents the control forces and moments applied by the AUV. $\tau_{d}$ is the disturbance vector composed of model parameter uncertainties and unknown environmental disturbances. When considering only motion in the horizontal plane, other irrelevant state variables are set to zero, resulting in a three-degree-of-freedom horizontal-plane model with surge velocity u, sway velocity v, and yaw rate r as state variables.

The propulsion system of the underactuated AUV studied in this paper consists of one propeller and two pairs of rudders. The horizontal rudders are used to control pitch and depth. By deflecting the rudder surfaces, they generate hydrodynamic lift or downforce, thereby changing the vehicle’s pitch angle for diving or surfacing. The vertical rudder is responsible for controlling the horizontal heading; its deflection generates lateral forces, driving the vehicle to turn around the vertical axis.

Based on the actual physical model of the propeller and hydrodynamic analysis, the thrust T and torque Q generated by the propeller can be calculated as [27]:

(3) $$\{\begin{array}{c} T=K_{T}\rho n_{p}^{2}D_{p}^{4}\\ Q=K_{Q}\rho n_{p}^{2}D_{p}^{5} \end{array}$$

where ρ denotes the density of the underwater environment fluid; $n_{p}$ is the actual rotational speed of the propeller; $D_{p}$ is the actual diameter of the propeller; $K_{T}$ is the thrust coefficient; $K_{Q}$ is the torque coefficient. The forces L and moments R generated by the rudders can be expressed as [27]:

(4) $$\{\begin{array}{c} L=\frac{1}{2}\rho C_{L}Au_{r}^{2}\\ R=\frac{1}{2}x_{rudder}\rho C_{L}Au_{r}^{2} \end{array}$$

where $C_{L}$ is the lift coefficient, which can be obtained by fitting the rudder angle δ; A is the area of the rudder; $x_{rudder}$ is the distance between the rudder and the axis. Overall, the AUV achieves motion state control and regulation by sending commands to adjust the propeller’s rotational speed $n_{p}$ and the two pairs of rudder angles $\delta_{r}$, $\delta_{s}$.

### 2.2. Path-Following Model

As mentioned above, tracking a 3D path in the o-xyz plane depends on the vertical and horizontal rudders and propeller thrust; tracking a 2D path requires one rudder and the propeller. The transition from 2D to 3D only adds control of the other rudder. Since the rudders and the propeller are mutually independent and uncoupled, the 3D path-following model can be viewed as an extension of the 2D case [28]. In this paper, theoretical analysis and algorithm verification are mainly based on the 2D plane, with conclusions extended to 3D. In the path-following problem, the core error quantities defining path deviation are the horizontal distance (i.e., cross-track error) and the deviation angle (i.e., heading error), which together define the pose deviation of the controlled object relative to the desired path. In the earth-fixed coordinate system xoy, the cross-track error $\varepsilon_{xy}$ is defined as the distance from the AUV’s current center of mass to the projection point on the reference path:

(5) $$\varepsilon_{xy}=\left(y_{0}-y_{ref}\right)\cos\alpha -\left(x_{0}-x_{ref}\right)\sin\alpha$$

where $\left(x_{0},y_{0}\right)$ are the coordinates of the AUV’s current center of mass, $\left(x_{ref},y_{ref}\right)$ are the coordinates of the projection point from the AUV’s center of mass onto the predefined trajectory, and α is the angle between the forward direction of the predefined trajectory and the positive y-axis. The heading angle ψ is defined as the angle between the AUV’s current forward direction and the positive y-axis.

**Figure 2:**
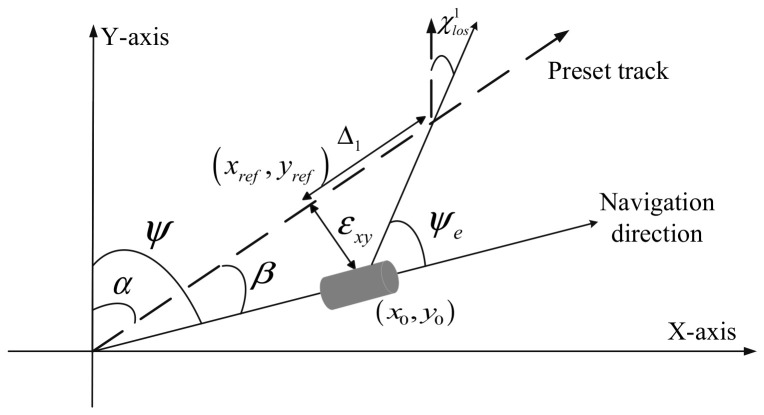
Schematic diagram of the ILOS algorithm in the xoy plane.

A schematic diagram of path following in the earth-fixed coordinate system xoy is shown in Figure 2. The heading error angle β is defined as the angle between the AUV’s current heading angle and the tangent direction of the reference path at the closest point. It reflects the dynamic error between the AUV’s attitude and the desired direction of travel and can be expressed as:

(6) β=ψ−α

The line-of-sight (LOS) guidance law is a geometric-based guidance strategy. It guides the vehicle toward a look-ahead point placed on the desired path, aiming to reduce the minimum distance between the vehicle’s position and the path to zero. To mitigate underwater disturbances such as currents during AUV navigation, this paper adopts an improved line-of-sight guidance law with an integral term. The core improvement is the introduction of an integral term for the cross-track error into the guidance law, compensating for disturbance errors by accumulating historical deviations [29,30]. Let the distance from the AUV’s current projection point to the look-ahead point be defined as the look-ahead distance $\Delta_{1}$. The horizontal-plane ILOS azimuth angle is then expressed as:

(7) $$\chi_{los}^{1}=\alpha -\arctan\left(\frac{1}{\Delta_{1}}\left(\varepsilon_{xy}+\frac{1}{T_{i}}\int_{0}^{t}\varepsilon_{xy}\left(t\right)dt\right)\right)$$

where the arctan function provides proportional compensation for the cross-track error, and $T_{i}$ is the integral time constant. The integral term serves to counteract the accumulated cross-track error $\varepsilon_{xy}$, ultimately achieving zero steady-state error. The look-ahead distance $\Delta_{1}$ adjusts the convergence rate: when the error is large or during sharp turns, decreasing $\Delta_{1}$ helps eliminate the error quickly; when the error is small or during straight-line cruising, increasing $\Delta_{1}$ allows the AUV to cruise more steadily and efficiently.

Consequently, the heading error angle relative to the LOS azimuth angle is defined as:

(8) $$\psi_{e}=\psi -\chi_{los}^{1}=\beta +\arctan\left(\frac{1}{\Delta_{1}}\left(\varepsilon_{xy}+\frac{1}{T_{i}}\int_{0}^{t}\varepsilon_{xy}\left(t\right)dt\right)\right)$$

where ψ is the current AUV heading. The heading error angle $\psi_{e}$ and cross-track error $\varepsilon_{xy}$ encapsulate the positional and attitudinal deviation of the AUV from the predefined path at the current moment. When extending this model to three dimensions, it is equivalent to projecting the predefined path onto the xoy plane and the xoz plane. In addition to the heading error angle $\psi_{e}$ and cross-track error $\varepsilon_{xy}$, a pitch error angle $\theta_{e}$, an associated error $\varepsilon_{xz}$, a look-ahead distance xoz, and an azimuth angle $\Delta_{2}$ in the $\chi_{los}^{2}$ plane are defined.

The control objective for AUV path following can be summarized as follows: by controlling the propeller’s rotational speed and adjusting the angles of the two pairs of rudders according to Equations (1) and (2), the AUV tracks the predefined path. This process fully reflects the strong nonlinearity of AUV motion underwater and the influence of current disturbances. Furthermore, this paper also accounts for model parameter uncertainties and unknown environmental disturbances.

## 3. Path-Following Control Algorithm Design

### 3.1. Fundamentals of Reinforcement Learning

Deep reinforcement learning (DRL) enables an agent to learn optimal policies through interaction with the environment, with its core being the Markov Decision Process (MDP) [14]. An MDP describes sequential decision-making problems through the tuple (S,A,P,R,γ), where S denotes the state space, representing all possible environmental states; A denotes the action space, representing all actions executable by the agent; γ is the discount factor, used to balance the importance of immediate versus future rewards; P is the state transition function; and R is the reward function. P and R satisfy the following relationships:

(9) $$P\left(s^{\prime }|s,a\right)=P[S_{t+1}=s^{\prime }|S_{t}=s,A_{t}=a]$$

(10) $$R\left(s,a\right)=E\left[R_{t+1}|S_{t}=s,A_{t}=a\right]$$

The agent’s goal is to find a policy π(a|s) (a mapping from states to actions) that maximizes the expected cumulative discounted reward:

(11) $$G_{t}=\sum_{k=0}^{\infty }\gamma^{k}R_{t+k+1}$$

We formulate the AUV path-following problem as a Markov Decision Model.

**First, state space design:** The algorithm needs to utilize current AUV state information and error information updated by the ILOS algorithm. In the 2D plane, the state vector is defined as:

(12) $$s_{t}={\left[\varepsilon_{xy},\sin\left(\psi_{e}\right),\cos\left(\psi_{e}\right),R,u_{r},v_{r},\sin\left(\psi \right),\cos\left(\psi \right),v_{c}^{x},v_{c}^{y}\right]}^{T}$$

where $u_{r},v_{r},\psi$ is the AUV state information, using state variables relative to the current; $\left[v_{c}^{x},v_{c}^{y}\right]$ is the current magnitude; $\varepsilon_{xy}$ is the cross-track error from the AUV’s current position to the target path; and $\psi_{e}$ is the current heading error angle. ψ and $\psi_{e}$ are expressed in trigonometric form to confine the two angles to the $\left(-1,1\right)$ interval. The look-ahead distance $\Delta_{1}$ is represented by the parameter $R_{1}$ and a constant $\Delta_{base}$, using a linear scaling as in Equation (13) to ensure $\Delta_{1}$ always stays within a physically reasonable range:

(13) $$\Delta_{1}=\Delta_{base}\cdot R_{1}$$

Other state variables are also normalized to facilitate neural network training. In the subsequent algorithm design, the state variables are divided into AUV state quantities and current vector quantities.

**Second, action space design:** The action space is designed as:

(14) $$a_{t}={\left[\delta_{r},n_{p},R_{1}\right]}^{T}$$

representing the vertical rudder angle (degrees), propeller speed, and look-ahead distance parameter. To avoid training oscillation or violating the Markov property, the look-ahead distance parameter $R_{1}$ obtained at time step t is used for the next step’s computation and does not appear directly in the state variables.

Similarly, the state and action spaces for 3D path following are expressed as Equations (15) and (16), where $R_{2}$ is the look-ahead distance parameter in the vertical plane.

(15) $$s_{t}^{\prime }={\left[\varepsilon_{xy},\varepsilon_{xz},\cos\left(\psi_{e}\right),\cos\left(\theta_{e}\right),R_{1},R_{2},u_{r},v_{r},w_{r},\cos\left(\psi \right),\cos\left(\theta \right),v_{c}^{x},v_{c}^{y},v_{c}^{z}\right]}^{T}$$

(16) $$a_{t}^{\prime }={\left[\delta_{r},\delta_{s},n_{p},R_{1},R_{2}\right]}^{T}$$

**Finally, reward function design:** In the 2D problem, the primary considerations are the key path-following metrics: cross-track error $\varepsilon_{xy}$ and heading error angle $\psi_{e}$. The smaller these two errors, the more accurate the path following. To ensure motion stability, the vertical rudder angle $\delta_{r}$ should not change too rapidly, and the vehicle speed V should remain stable near a desired value. Therefore, the reward function in this paper includes these four terms and is designed as:

(17) $$r=\left\{\begin{array}{c} \rho_{\varepsilon }\cdot e^{-k_{\varepsilon }\cdot \left|\varepsilon_{xy}\right|}+\rho_{\psi }\cdot e^{-k_{\psi }|\psi_{e}|}+\rho_{\delta }\cdot {\left|\delta_{r}^{t}-\delta_{r}^{t-1}\right|}^{2}+\rho_{v}\cdot |V-V_{s}{|}^{2},\;if\;|\psi_{e}|<\pi /2\\ \rho_{\varepsilon }\cdot e^{-k_{\varepsilon }\cdot \left|\varepsilon_{xy}\right|}+\rho_{\psi }\cdot -e^{-k_{\psi }\left(|\psi_{e}|-\pi \right)}+\rho_{\delta }\cdot {\left|\delta_{r}^{t}-\delta_{r}^{t-1}\right|}^{2}+\rho_{v}\cdot |V-V_{s}{|}^{2},\;if\;|\psi_{e}|\geq \pi /2 \end{array}\right.$$

where the ρ series are weight coefficients, and $\rho_{\varepsilon },\rho_{\psi },\rho_{\delta },\rho_{v}$ are all negative constants, encouraging the agent to learn towards high accuracy and stability. $V_{s}$ is the desired AUV speed, and $k_{\varepsilon }$ and $k_{\psi }$ penalize cross-track error and heading error, respectively. Extending the 2D problem to 3D, we additionally consider the pitch error angle $\theta_{e}$ and error $\varepsilon_{xz}$ in the xoz plane, defined similarly to Equation (32), with new penalty coefficients $k_{\varepsilon }^{z}$ and $k_{\theta }$, and weight coefficient $\rho_{\varepsilon }^{z},\rho_{\theta }$. To avoid too many reward terms, the change in rudder angle penalty is subtracted.

### 3.2. Path-Following Control Algorithm Design

In reinforcement learning, the fundamental difference between model-based and model-free algorithms lies in whether the agent explicitly uses and relies on a dynamics model of the environment during learning or decision-making. Since the accurate environmental model for the AUV path-following problem is difficult to obtain, and collecting samples on a real AUV is extremely costly and risky, this paper adopts a model-free reinforcement learning algorithm. Because model-based methods typically have relatively lower sample efficiency and often require a large amount of interaction data to converge, which would be disastrous for physical AUV experiments, we choose to first train the agent in a simulation environment and then transfer it from simulation to physical experiments.

**Figure 3:**
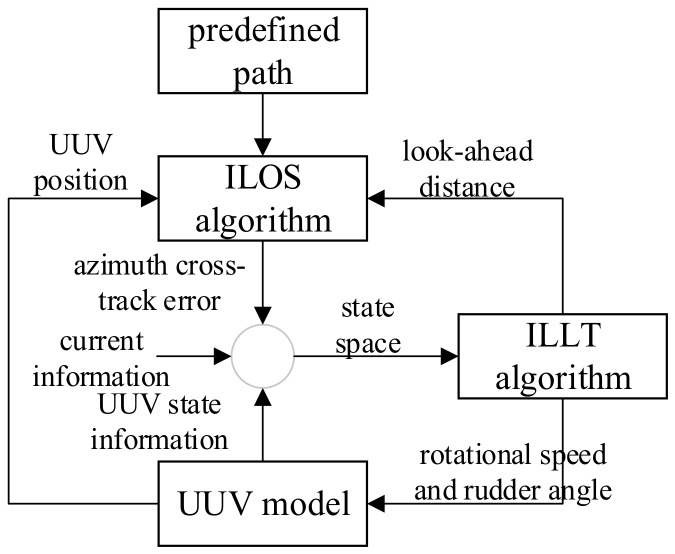
Schematic diagram of the control process.

We employ a model-free reinforcement learning framework and propose the ILLT (Improved LOS-LSTM-TD3) algorithm. The schematic of the control algorithm acting on the AUV path-following model is shown in Figure 3.

At the current time step, the ILOS algorithm computes the current azimuth angle $\chi_{los}$ and cross-track error $\varepsilon_{xy}$ based on the AUV’s real-time position information, predefined path information, and look-ahead distance. These are combined with the AUV’s state information to form the state space for the control algorithm. The control algorithm outputs the AUV’s rotational speed $n_{p}$, horizontal rudder angle $\delta_{s}$, vertical rudder angle $\delta_{r}$, and the look-ahead distance. The first three serve as input commands for the AUV, which updates its motion state. The new motion state, along with the look-ahead distance and the predefined path, are fed back into the ILOS algorithm, completing the computation for the current time step and proceeding to the next.

The ILLT algorithm is based on the currently state-of-the-art TD3 algorithm. TD3 adopts the Actor–Critic framework [28], consisting of one Actor network, two Critic networks, and their corresponding target networks. The Actor network learns an optimal policy and outputs actions; the Critic network evaluates action values. The Actor’s update direction depends on the Critic’s evaluation, adjusting parameters towards larger Q-values. Experiences generated by the Actor through environment interaction are stored in a replay buffer, from which the Critic samples for learning and continuously refines its evaluation criteria.

Based on the existing TD3 network structure and tailored to the practical underwater AUV path-following scenario, the ILLT algorithm incorporates the following major improvements:

**1. Progressive unfreezing strategy** with a feature extractor and a current encoder in the input layer. The progressive unfreezing strategy is applied to training from the source domain to the target domain, aiming to accelerate network training under different current environments. The goal of the feature extractor is to extract deep temporal dynamic features from a historical window of the AUV’s own states; these features should reflect only the AUV’s dynamics and be decoupled from external environmental factors such as currents, so that the output feature vector f does not vary with currents. The current encoder processes only current information, focusing on extracting current features. After obtaining the current AUV state features and current features, a feature-level linear modulation technique (citation) is employed, using an attention network to dynamically fuse the two. A scalar weight is dynamically computed to control the contribution strength of current information to the final feature, and the two are fused. By feeding the input state quantities separately into the feature extractor and the current encoder, the model can, during parameter training, adapt to changes in currents across different environments by gradually releasing the trainability of different network modules according to a schedule. This allows the model to first leverage the AUV’s dynamic features to quickly learn how to modulate features based on the current, and then progressively adjust deeper representations, thereby avoiding catastrophic forgetting and effectively balancing the retention of source-domain knowledge with rapid adaptation to the target domain. The specific progressive unfreezing strategy is detailed in Table 1 later.

**2. Capturing temporal dependencies** by introducing an LSTM structure in the input layer. The ILOS computation already includes historical information of the cross-track error (integral term), which effectively compensates for constant disturbances and drives the steady-state error to zero, but it cannot capture temporal dependencies of other states (e.g., velocity, angular velocity, attitude). The introduction of the LSTM structure effectively learns the dynamic variation patterns among multi-dimensional states and captures the temporal dependencies (e.g., inertia, velocity trends) of the AUV’s own state sequence. The functions of the LSTM structure and the ILOS integral term overlap but are not identical.

Based on the above improvements, the network architecture of the proposed ILLT algorithm is as follows. The algorithm contains one Actor network, two Critic networks, and their corresponding target networks. In each network structure, the input layer consists of a feature extractor and a current encoder.

In the feature extractor, the state quantities are:

(18) $$s_{t}^{1}={\left[\varepsilon_{xy},\sin\left(\psi_{e}\right),\cos\left(\psi_{e}\right),R,u_{r},v_{r},\sin\left(\psi \right),\cos\left(\psi \right)\right]}^{T}$$

**Figure 4:**
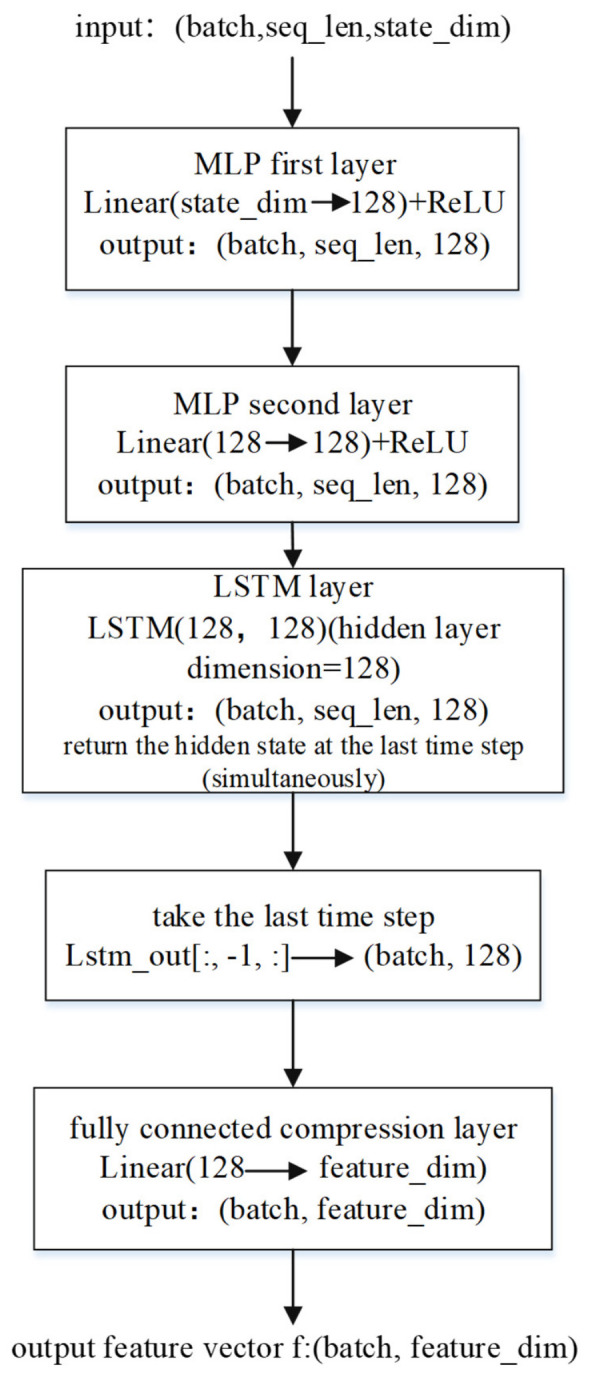
Schematic diagram of the feature encoder structure.

First, two fully connected layers map the low-dimensional raw state at a single time step to a high-dimensional hidden space to enhance feature representation. Then, consecutive k (here set to 4) time steps of state data $\left(s_{t-k-1}^{1},\cdots ,s_{t}^{1}\right)$ are fed into an LSTM network to capture temporal dependencies and learn the hidden characteristics of AUV motion and the predefined path. Finally, a feature transformation layer (feature_dim = 64) yields the output vector f. The structure of the feature encoder and the dimensions of each layer are shown in Figure 4. The hidden layer dimension of the LSTM network is 64.

The state quantities for the current encoder are:

(19) $$s_{t}^{2}={\left[v_{c}^{x},v_{c}^{y}\right]}^{T}$$

which is designed as a simple two-layer MLP. The first layer changes the three-dimensional current velocity to 32 dimensions, and the second layer changes from 32 to 64 dimensions, yielding the current output vector e, which has the same dimension as the feature encoder output f.

The core of the attention fusion module is to dynamically modulate the current vector e and fuse it with the feature f. First, the concatenated vector $\left[f;e\right]$ is passed through a full linear layer of the same dimension (128), then through two fully connected layers, first reducing to 64 dimensions and then to a single output feature, which is passed through a Sigmoid activation function to obtain the coefficient α. Essentially, α learns a soft gating switch that determines the influence of current information on the main features. The projected current embedding is multiplied by the attention weight and then added to the original feature to obtain the final feature:

(20) $$f_{fused}=f+\alpha \cdot e_{proj}$$

The Actor and Critic networks use separate feature extractors, current encoders, and feature-level linear modulation. In the Actor network, the modulated feature $f_{fused}$ is passed through a two-layer fully connected network, and finally an activation function converts the output to continuous actions. In the Critic network, the modulated feature $f_{fused}$ and the action α are concatenated along the feature dimension, and a two-layer fully connected network directly outputs the scalar Q value.

During network parameter training, a progressive unfreezing strategy is introduced. This training strategy effectively balances the retention of source-domain knowledge and rapid adaptation to the target domain in response to varying currents across environments. The updates of the feature extractor, current encoder, and attention fusion module are naturally achieved through backpropagation of the Actor and Critic losses. The Actor and Critic feature extractors are independent but share the same unfreezing schedule. In transfer learning, we control which modules participate in updates by setting parameter attributes. By gradually releasing the trainability of different modules according to the schedule, the model can first exploit the AUV’s dynamic features to quickly learn how to modulate features based on the current, then progressively adjust deeper representations, thereby avoiding catastrophic forgetting and accelerating convergence. The unfreezing training strategy is designed as shown in Table 1. In this paper, $\alpha_{1}$ and $\alpha_{2}$ are set to 0.4 and 0.8, respectively, depending on the specific task and network size.

In the TD3 algorithm, the Critic learns the state-action value function by minimizing the Bellman error. For each Critic $Q_{i}\left(i=1,2\right)$, at time step t, the loss is:

(21) $$L_{Q_{i}}=E\left[{\left(Q_{i}\left(s_{t},a|\theta^{Q_{i}}\right)-y\right)}^{2}\right]$$

where the target value y uses the minimum of the two target Critics to suppress overestimation:

(22) $$y=r+\gamma \underset{i=1,2}{min}Q_{i}^{\prime }\left(s_{t+1},\mu^{\prime }\left(s_{t+1}|\theta^{\mu }\right)|\theta^{Q^{i}}+\varepsilon \right)$$

where ε represents the target policy smoothing noise. This setting effectively handles the uncertainty in policy output.

The Actor is updated by maximizing the Q-value from Critic1:

(23) $$L_{actor}=-E\left[Q_{1}\left(s,\mu \left(s\right)\right)\right]$$

In this paper, on top of the above losses, a consistency loss and a regularization loss are added. To enhance policy robustness against observation noise, a consistency loss is added, forcing the Actor’s output actions for noisy inputs to be close to those for the original input. The consistency loss is defined as follows:

(24) $$L_{cons}=E_{s}\left[{\left\|\mu \left(s\right)-\mu \left(s+\delta \right)\right\|}^{2}\right],\;\;\;\delta ∼N\left(0,\sigma_{s}\right)$$

Since we retain the source-domain pre-trained feature extractor and gradually unfreeze it during transfer, it is necessary to prevent the feature extractor parameters from deviating excessively from the source domain. To avoid catastrophic forgetting, we apply L2 regularization, which only affects the currently trainable parameters of the feature extractor:

(25) $$L_{reg}=\lambda_{reg}\sum_{i\in trainable}{\left\|\theta -\theta^{source}\right\|}^{2}$$

where $\theta^{source}$ represents the saved parameter values from the end of the source-domain pre-training.

Therefore, the total loss for the Critic network is:

(26) $$L_{critic_{i}}^{total}=L_{Q_{i}}+\lambda_{reg}L_{reg_{i}}^{critic_{i}}$$

where $\lambda_{reg}$ represents the regularization weight, which gradually decreases as training progresses.

The total loss for the Actor network is:

(27) $$L_{actor}^{total}=L_{actor}+\lambda_{cons}L_{cons}+\lambda_{reg}L_{reg}^{actor}$$

Similar to the TD3 algorithm, the new algorithm employs a delayed Actor update strategy, updating the Actor network every d steps. Both the Actor and Critic have target networks with identical structures to their respective main networks, and parameter updates are performed using a soft update method:

(28) $$\theta_{t\arg et}\leftarrow \tau \theta +\left(1-\tau \right)\theta_{t\arg et},\tau \leq 1$$

In the 3D problem, the state quantities in the feature extractor and the current encoder are given by Equations (29) and (30), respectively, while other network parameter structures remain the same as in the 2D case.

(29) $$s_{t}^{1^{\prime }}={\left[\varepsilon_{xy},\varepsilon_{xz},\cos\left(\psi_{e}\right),\cos\left(\theta_{e}\right),R_{1},R_{2},u_{r},v_{r},w_{r},\cos\left(\psi \right),\cos\left(\theta \right)\right]}^{T}$$

(30) $$s_{t}^{2^{\prime }}={\left[v_{c}^{x},v_{c}^{y},v_{c}^{z}\right]}^{T}$$

Taking the 2D path-following problem as an example, the parameter counts of each module in the ILLT algorithm are: feature encoder 46,592; current encoder 2208; attention fusion module 12,481; policy head 4355; Q-head 8833; the total parameter count of the ILLT algorithm is 205,864. The standard TD3 algorithm (256-dim) has a total of 208,645 parameters. Similarly, in the 3D problem, the total parameters of ILLT are 207,754, and standard TD3 (256-dim) is 213,255. In both dimensions, the ILLT algorithm has parameter counts close to those of standard TD3, but gains key capabilities such as temporal modeling and dynamic current modulation.

In summary, the ILLT algorithm flow includes source-domain pre-training and target-domain transfer training, as shown in Table 2 and Table 3.

## 4. Simulation Experiments

### 4.1. Simulation Model Construction

**Figure 5:**
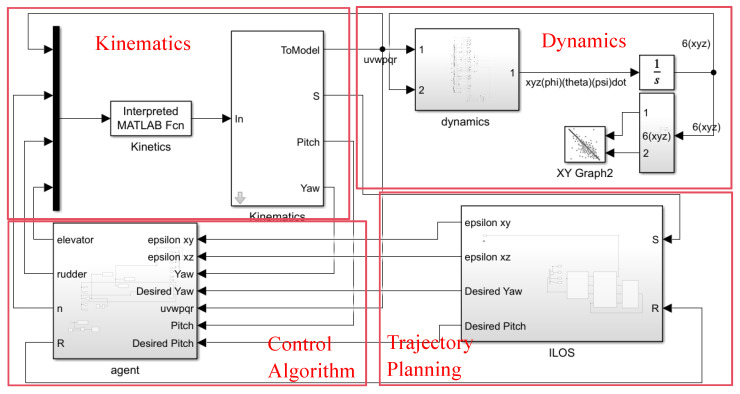
Simulink simulation model diagram.

The two-dimensional AUV path-following model based on deep reinforcement learning presented in this paper uses Python as the algorithm programming tool and Matlab (Matlab 2024b) as the platform for building the AUV environment, achieving Python–Matlab co-simulation. This model mainly consists of four modules: the Kinematics Module (AUV Kinetics Module), the Dynamics Module (AUV Dynamics Module), the Control Algorithm Module (DRL Controller Module), and the Path Planning Module (ILOS Path Planning Module). The functional modules are shown in Figure 5.

The Kinematics Module and Dynamics Module implement the kinematic and dynamic equations from Section 2.1, respectively. The Trajectory Planning Module uses the ILOS algorithm based on the predefined path and AUV state information to obtain the heading error angle and cross-track error, implementing the path-following model from Section 2.2. The Control Algorithm Module provides intelligent decision-making for the AUV. The ILLT algorithm designed in this paper is implemented using the PyTorch framework in Python, and the control algorithm interacts with the Matlab/Simulink model via the Matlab Engine API, outputting control parameters through the Python program, thus implementing the control algorithm design from Section 3. In actual computation, the key parameters of the ILLT algorithm are set as shown in Table 4. Standardized test scenarios are essential for quantitative evaluation of control algorithm performance; Ref. [31] constructed a case test library for autonomous vehicle obstacle avoidance validation, providing a unified algorithm evaluation framework, which informs the design of our 2D and 3D path-following simulation comparison experiments. Some parameter settings refer to [28] and [32].

### 4.2. Attention Coefficient Computation

According to theoretical analysis, the attention coefficient α should be a dynamic gating value between 0.1 and 0.9, capable of adaptively reflecting the importance of currents to the current control decision. Therefore, taking the 2D environment as an example, we observe the variation in the attention coefficient α under different current magnitudes. Due to the long simulation time, six current magnitudes were set, and the simulation results are shown in Table 5.

From Table 5, the attention coefficient α is positively correlated with current magnitude: the stronger the current, the larger the attention coefficient. When the current is small ($V_{c}={\left[0.1\;m/s,-0.1\;m/s\right]}^{T}$), the attention coefficient is 0.12, close to 0; when the current reaches $V_{c}={\left[2\;m/s,2\;m/s\right]}^{T}$, where the absolute speed approaches the AUV’s maximum speed, the attention coefficient is close to 1, reaching 0.91.

### 4.3. Two-Dimensional Plane Simulation Experiments

Based on the preceding analysis, simulations are conducted for training from the source domain to the target domain. To evaluate algorithm performance, the proposed ILLT algorithm is compared against the ILT (Improved LOS-TD3) algorithm without the LSTM network, the LLT (LOS-LSTM-TD3) algorithm without training the look-ahead distance, and traditional PID control. A predefined path is constructed in Matlab, with the path expression given by Equation (31):

(31) $$y=\{\begin{array}{cc} 0.28x+1; & \;0\leq x\leq 40\\ -0.21x+20.6; & \;40<x\leq 64\\ 0.5x-25; & \;64\leq x<80 \end{array}$$

During source-domain training, the current in the environment is set to zero, and the disturbance matrix $\tau_{d}={\left[0.2,0.2\right]}^{T}$ is set randomly. Source-domain pre-training is conducted. Different algorithms are run for five independent repeated experiments with five different random seeds. The control algorithm is trained with 500 episodes during the learning, and for each episode, the training step is limited to 450 timesteps.

**Figure 6:**
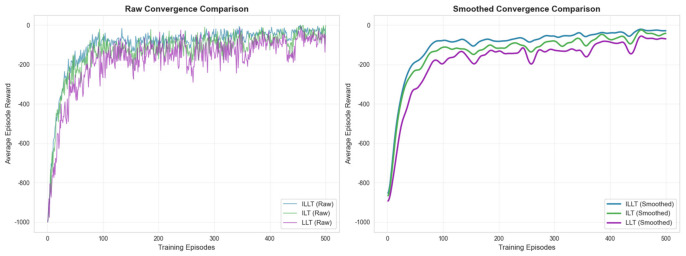
Reward value variation curves for the three algorithms.

The changes in reward values for the ILLT, ILT, and LLT algorithms are compared. Figure 6 shows the average reward values across the five experiments for the different algorithms. The left panel shows the reward value for each episode, and the right panel shows the average value over five consecutive episodes. At the initial stage of iterations, all three algorithms have low reward values. As the number of iterations increases, the reward values converge to near 0, with the ILLT algorithm’s reward value closest to 0, indicating better convergence performance. Within the first 0–40 iterations, the ILLT algorithm does not show a significant advantage, likely due to its larger network requiring some initial training time. The ILLT algorithm exhibits faster convergence speed between 40 and 100 iterations and converges to a good value around 100 iterations, whereas the ILT and LLT algorithms require approximately 200 and 250 iterations, respectively, to achieve comparable convergence. Therefore, the ILLT algorithm can be considered to have faster convergence speed.

**Figure 7:**
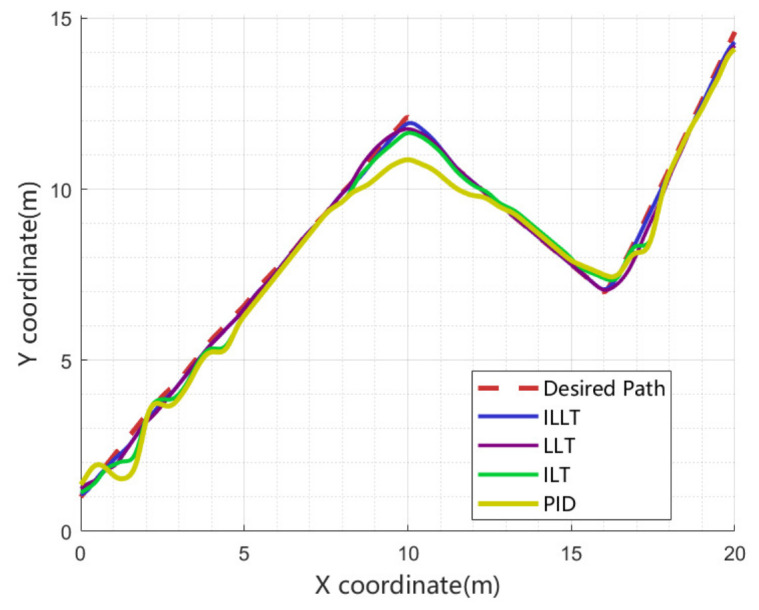
Comparison of motion trajectories for different methods.

**Figure 8:**
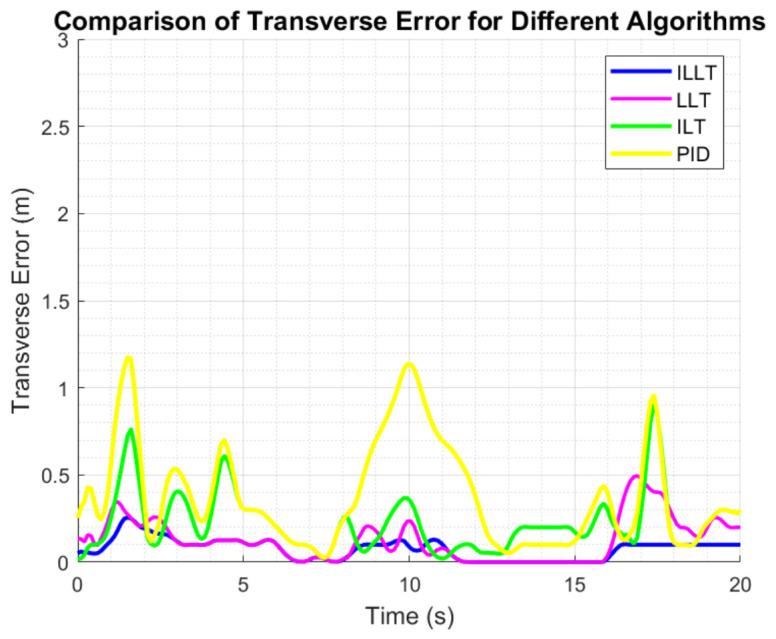
Comparison of cross-track error over time.

The results of path following and cross-track error are compared, as shown in Figure 7 and Figure 8. In the figures, the red dashed line represents the predefined path, while the blue, purple, yellow, and green solid lines represent the ILLT, LLT, ILT, and PID control algorithms, respectively.

The simulation results in Figure 7 and Figure 8 show that all four algorithms can achieve basic tracking. All four algorithms converge quickly during straight-line segments, with the proposed ILLT algorithm showing the fastest convergence speed. At path turning points, all algorithms exhibit some fluctuation due to the change in the path’s trend. However, the trajectory under the ILLT algorithm remains closest to the predefined path, indicating that the training of the look-ahead distance and the introduction of the LSTM network effectively enable prediction and estimation of the predefined path. Therefore, the proposed ILLT algorithm demonstrates the highest accuracy in tracking the predefined path, exhibiting optimal tracking performance. To further analyze the tracking performance of the four algorithms, key data from path following are summarized in Table 6.

In Table 6, compared to the LLT algorithm, the ILLT algorithm reduces the maximum and average cross-track errors by 60.2% and 60%, respectively. Compared to the ILT algorithm, the reductions are 47.8% and 48.5%, respectively. Compared to PID control, the reductions are 50.4% and 66.4%, respectively, indicating that the ILLT algorithm achieves high path following accuracy. The maximum reward value is 6.6 and 4.4 higher than the LLT and ILT algorithms, respectively. At 100 iterations, the average reward values for the three algorithms represent 92.1%, 87.2%, and 79.5% of their respective maximum rewards, suggesting that the proposed ILLT algorithm has faster convergence speed.

**Figure 9:**
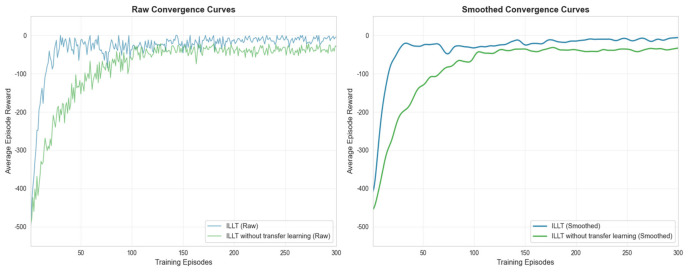
Reward value variation curves for two-dimensional path target-domain transfer training.

To validate the algorithm’s transfer characteristics, under the same predefined path conditions, the current in the environment is set to $V_{c}={\left[0.3\;m/s,-0.1\;m/s\right]}^{T}$, and the disturbance matrix $\tau_{d}={\left[0.1,0.3\right]}^{T}$ is set. We compare the proposed ILLT algorithm (using the transfer strategy of Table 3) with a control algorithm that directly copies the source-domain pre-trained parameters to the target domain without the progressive unfreezing strategy, i.e., all networks are trained simultaneously in the target domain. This experiment aims to validate the effectiveness of the progressive unfreezing strategy. Again, five independent trials with different seeds are performed, and the average reward curves are shown in Figure 9.

In Figure 9, the ILLT algorithm exhibits better initial values and converges to a good reward value of −30 around the 25th iteration, while the comparison algorithm (LOS-TD3) requires about 100 iterations to converge to −50. Compared with source-domain training in Figure 6, where about 90 iterations were needed to reach a reward of −90, the improvement to −30 requires about 400 iterations. ILLT shows faster convergence in the target domain, validating the effectiveness of the progressive unfreezing transfer strategy.

### 4.4. Three-Dimensional Plane Simulation Experiments

**Figure 10:**
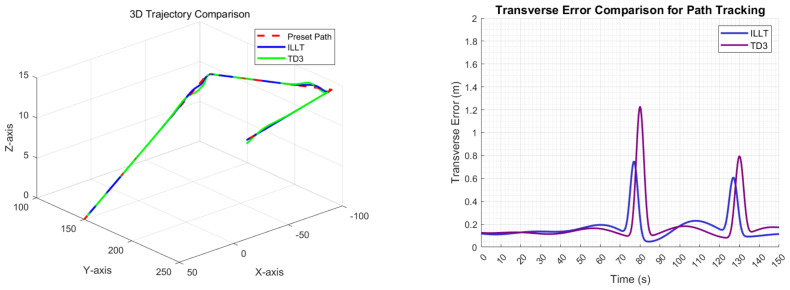
Results of three-dimensional path following and cross-track error.

To verify the effectiveness of the ILLT algorithm in three-dimensional path following, a three-dimensional predefined path is first constructed in Matlab with no current in the environment, and source-domain pre-training is conducted. The TD3 algorithm is used as a baseline for comparison. The results of three-dimensional path following and cross-track error are shown in Figure 10.

From Figure 10, it can be observed that in a three-dimensional environment, the proposed ILLT algorithm still achieves higher tracking accuracy compared to the TD3 algorithm.

**Figure 11:**
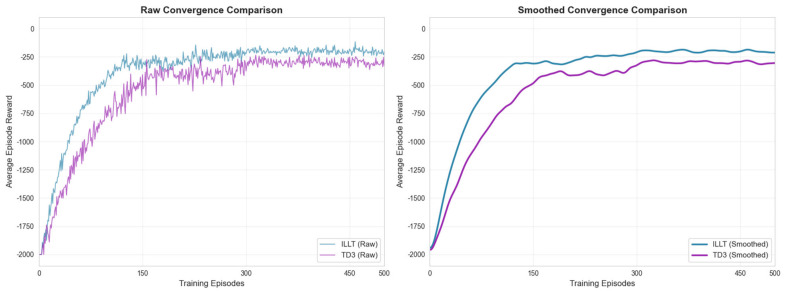
Reward value variation curves for the two algorithms.

The changes in reward values during network training for ILLT and TD3 algorithms are compared. Figure 11 shows the average reward values across five experiments for the different algorithms. The left panel shows the reward value for each episode, and the right panel shows the average value over five consecutive episodes.

From the figure, it is evident that under similar predefined path conditions, due to the higher complexity of the three-dimensional space compared to the two-dimensional case, more iterations are needed to converge to an optimal value. However, the ILLT algorithm converges significantly faster than the TD3 algorithm.

**Figure 12:**
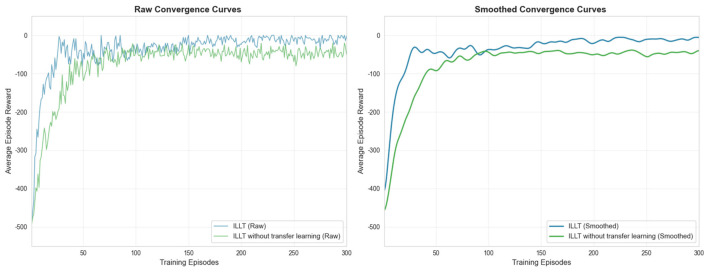
Reward value variation curves for three-dimensional path target-domain transfer training.

To verify the algorithm’s transfer characteristics, under the same predefined path conditions, the current in the environment is set to $V_{c}={\left[0.2\;m/s,0.2\;m/s,-0.1\;m/s\right]}^{T}$, and the disturbance matrix $\tau_{d}={\left[0.3,0.1,0.2\right]}^{T}$ is set. The proposed ILLT algorithm is compared against an algorithm without the transfer strategy, i.e., the ILLT algorithm using the transfer strategy in Table 3, while the control algorithm directly copies the source-domain pre-trained network parameters to the target domain for training. Again, five independent repeated experiments are conducted with five different random seeds. The convergence curves of the reward function are shown in Figure 12. Clearly, ILLT converges faster, confirming the effectiveness of the progressive unfreezing transfer strategy in the 3D case.

## 5. Physical Experiments

To further validate the practical applicability of the ILLT algorithm, underwater physical experiments are conducted. Since high-precision GPS positioning is difficult to achieve underwater, and makes it difficult to analyze algorithm performance more comprehensively, two types of experiments—tank and lake—are carried out based on different measurement principles, fully considering experimental measurement errors. In the tank, the water quality is good and shallow; the AUV relies on visual positioning to track a predefined path laid on the tank bottom. This experiment involves constant-depth motion, validating 2D path following. In the lake, the water is deeper and of lower quality; the AUV uses an integrated navigation system to validate 3D path following.

The AUV is equipped with various sensors (velocity, acceleration, etc.) that provide noisy velocity information. The upper control layer uses an RK3588 chip to implement complex control functions, including the ILLT algorithm and image processing; control commands are sent to the lower-level microcontroller for actuator implementation. The AUV is also connected to a shore-based computer via a cable.

### 5.1. Tank Path-Following Experiment

**Figure 13:**
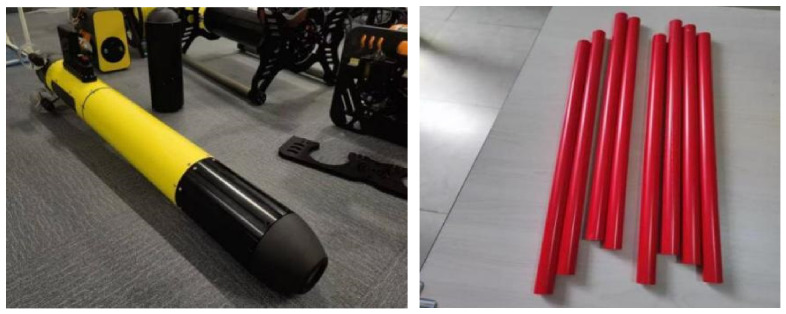
Physical AUV and PVC pipes.

The experiment was conducted in an artificial tank of size 25 m × 15 m × 1.5 m (depth), with clear water and a water flow control system. For data analysis, the AUV performed constant-depth motion. The predefined path was laid on the tank bottom using PVC pipes. Photos of the AUV and PVC pipes are shown in Figure 13.

**Figure 14:**
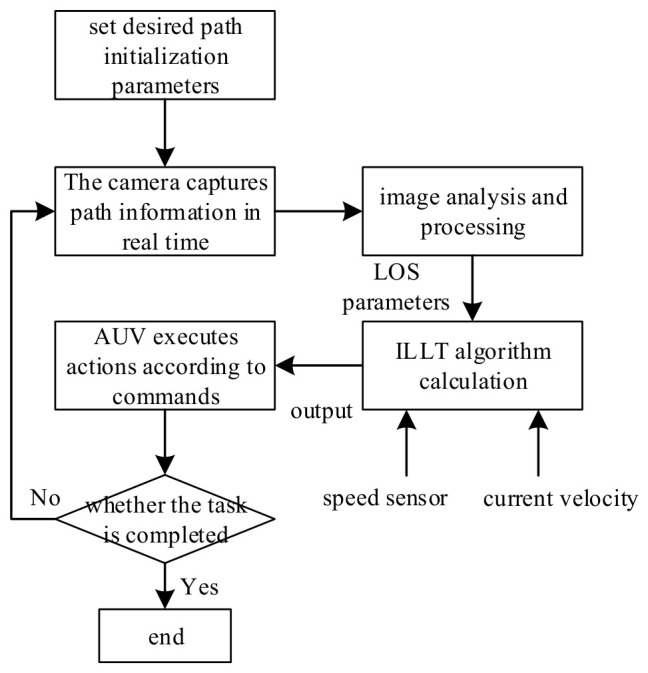
AUV workflow diagram.

The main workflow of the AUV is illustrated in Figure 14.

In the experiment, the time step was 0.1 s. Images captured by the camera were sampled at 0.1 s intervals; image analysis (as described in Section 5.1) took approximately 0.03 s. Then, the ILLT algorithm computed the outputs, taking about 0.02 s on average. Thus, the total delay from image acquisition to control output was about 0.05 s, which is negligible for the control process.

**Figure 15:**
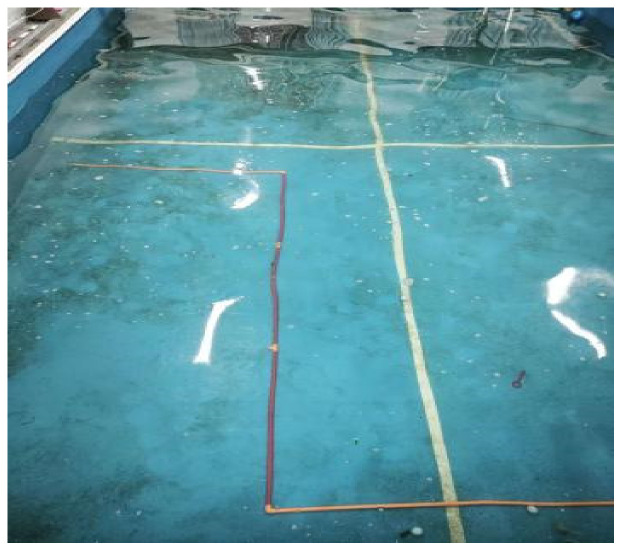
Overview of the predefined trajectory.

**Figure 16:**
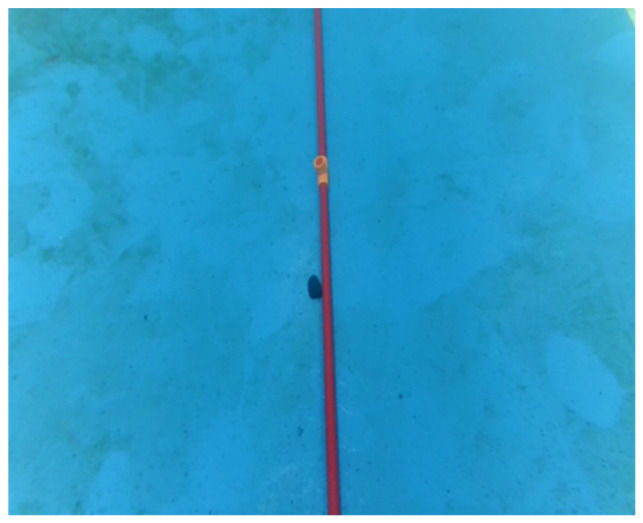
Image captured by the camera.

The predefined path was constructed using multiple PVC pipe segments connected at turning points, with the first segment 3 m, the second 6 m, and the third 3 m. The overall layout is shown in Figure 15, and a real-time camera image is shown in Figure 16.

#### 5.1.1. Image Analysis and Processing

Since the AUV obtains environmental information mainly through the camera, the images must be processed to extract useful LOS data as inputs to the ILLT algorithm. Underwater impurities require image pre-processing. We adopt a three-space feature enhancement network framework combining HSV, LAB, and RGB color spaces to obtain a set of discrete feature points {(x,y(x))} of the predefined path, which are then fitted to obtain the line segment function y(x)=k⋅x+b in the image coordinate system.

**Figure 17:**
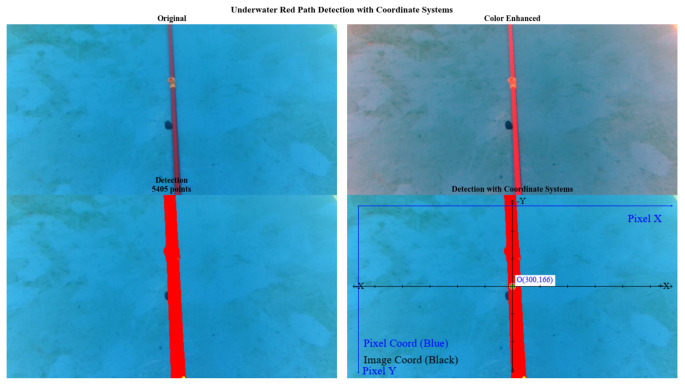
Underwater image processing comparison and coordinate system illustration.

The AUV uses a binocular camera with a downward tilt angle of 45°, baseline 40 mm, field of view 100°, resolution 1080p, and constant depth of 0.8 m above the tank bottom. The image coordinate system and pixel coordinate system are established, as shown in Figure 17.

To obtain the cross-track error $\varepsilon_{xy}$ and heading error angle β as inputs to the agent, the path function y(x)=k⋅x+b in the image coordinate system must be transformed to the world coordinate system. With the optical center as the origin and axes parallel to the image coordinate axes, and the Z-axis along the optical axis, we establish the camera coordinate system $O_{c}-X_{c}Y_{c}Z_{c}$. The world coordinate system $O_{w}-X_{w}Y_{w}Z_{w}$ is the real-world coordinate system of the camera. Based on the camera imaging principle, coordinates can be transformed between pixel and world coordinates:

(32) $$Z_{c}\left[\begin{array}{c} \begin{array}{c} u\\ v \end{array}\\ 1 \end{array}\right]=\left[\begin{array}{ccc} 1/dx & 0 & u_{0}\\ 0 & 1/dy & v_{0}\\ 0 & 0 & 1 \end{array}\right]\left[\begin{array}{cccc} \begin{array}{c} f\\ 0\\ 0 \end{array} & \begin{array}{c} 0\\ f\\ 0 \end{array} & \begin{array}{c} 0\\ 0\\ 1 \end{array} & \begin{array}{c} 0\\ 0\\ 0 \end{array} \end{array}\right]\left[\begin{array}{cc} R & T\\ 0^{T} & 1 \end{array}\right]\left[\begin{array}{c} X_{W}\\ Y_{W}\\ Z_{W}\\ 1 \end{array}\right]=L_{1}L_{2}\left[\begin{array}{c} X_{W}\\ Y_{W}\\ Z_{W}\\ 1 \end{array}\right]$$

(33) $$L_{1}=\left[\begin{array}{ccc} 1/dx & 0 & u_{0}\\ 0 & 1/dy & v_{0}\\ 0 & 0 & 1 \end{array}\right]\left[\begin{array}{cccc} \begin{array}{c} f\\ 0\\ 0 \end{array} & \begin{array}{c} 0\\ f\\ 0 \end{array} & \begin{array}{c} 0\\ 0\\ 1 \end{array} & \begin{array}{c} 0\\ 0\\ 0 \end{array} \end{array}\right]$$

(34) $$L_{2}=\left[\begin{array}{cc} R & T\\ 0^{T} & 1 \end{array}\right]$$

where dx,dy are the physical sizes of each pixel in the image coordinate axes, f is the focal length, $\left(u_{0},v_{0}\right)$ are the image center coordinates, $L_{1}$ is the intrinsic camera matrix determined by the camera components, $L_{2}$ is the extrinsic matrix determined by camera position and orientation, with R the rotation matrix and T the translation vector.

To obtain the intrinsic and extrinsic parameters, we calibrate the camera using Zhang’s method [33]. The final parameters are listed in Table 7.

After determining the intrinsic and extrinsic parameters, the path function y(x)=k⋅x+b in the image coordinate system is transformed to the world coordinate system function $y^{\prime }(x)=k^{\prime }\cdot x+b^{\prime }$. Then, the cross-track error $\varepsilon_{xy}$ and deviation angle β can be computed, and subsequently the heading error angle $\psi_{e}$ as input to the ILLT algorithm.

**Figure 18:**
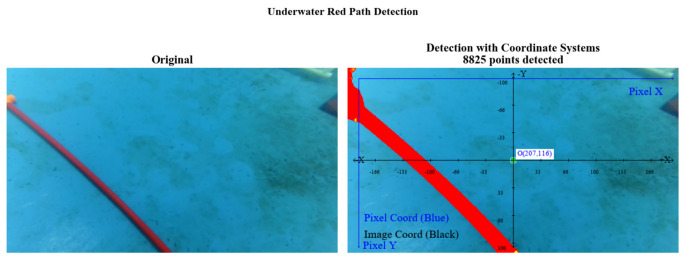
Image captured by the camera.

For example, an image captured at a certain moment is shown in Figure 18.

The three-space feature enhancement network framework first obtains a binary mask, then path feature extraction on the mask yields discrete points, and linear fitting produces the path function in the pixel coordinate system:

(35) y = 0.9613x + 35.01

Then, using the calibration parameters (Table 7), the image coordinates are transformed to world coordinates, yielding:

(36) $$Y_{w}=0.92X_{w}+0.44$$

Consequently, the cross-track error $\varepsilon_{xy}$ is 0.56 m, and the heading angle ψ is 51.6°.

#### 5.1.2. Physical Experiments

Following the previous analysis, the predefined path is first expressed mathematically, and source-domain training is performed in simulation with zero current. Then, target-domain (tank environment) experiments are conducted.

**Figure 19:**
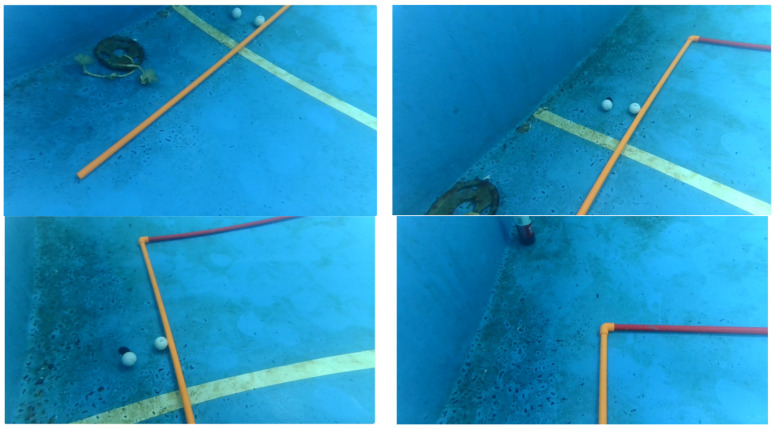
Initial stage.

**Figure 20:**
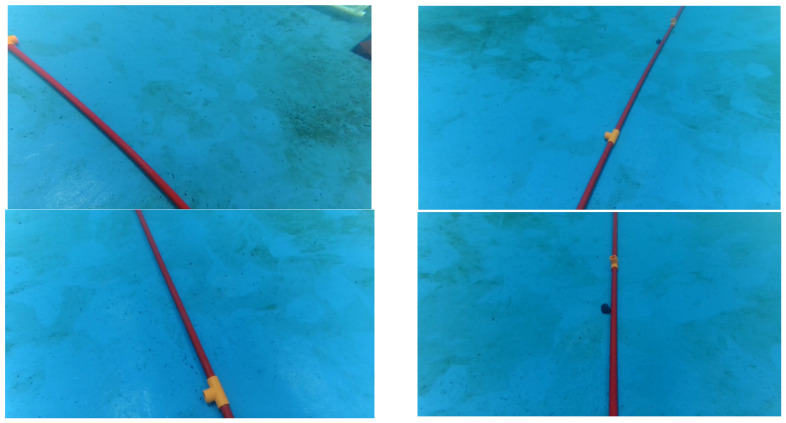
Turning stage.

**Figure 21:**
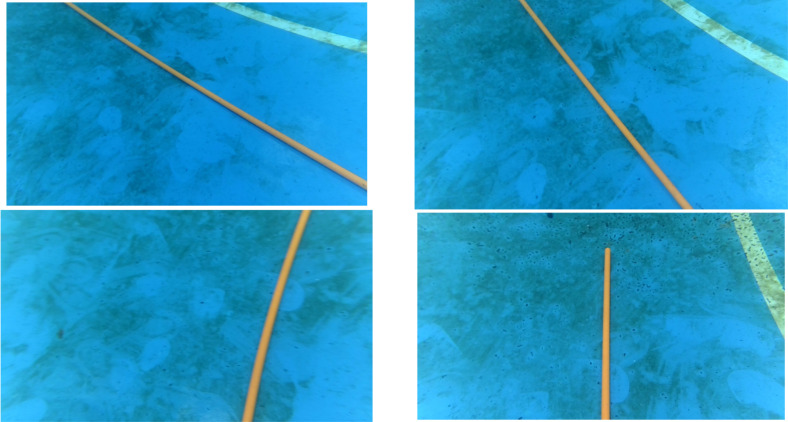
Final convergence stage.

Different current conditions were set, and 30 repeated trials were conducted under each condition, with different initial positions. Data from the camera and sensors were transmitted to the shore computer in real time for analysis. We illustrate with a current speed $\left[v_{c}^{x},v_{c}^{y}\right]=\left[0.3,0\right]\;m/s$. The entire tracking process can be divided into three phases: an initial phase, turning adjustment phase, and terminal straight-line phase. Figure 19, Figure 20 and Figure 21 show images from the 20th trial.

In the initial stage, the AUV first gradually accelerates from a distant starting position to the desired speed 1.5 m/s and subsequently maintains that speed stably. Due to the large initial pose deviation, brief oscillations appear in the control system, but tracking of the predefined path is quickly achieved.

During the turning adjustment stage, due to the abrupt change in the curvature of the predefined path at the bend, the AUV experiences significant cross-track position error and heading deviation. However, the control algorithm enables the AUV to quickly compensate for these deviations, rapidly converging and re-tracking the desired trajectory.

In the final convergence phase, the AUV quickly converged after the turn and completed the entire track, demonstrating efficiency and robustness. To quantify the tracking results, we computed the average cross-track error across trials under different current conditions and compared the maximum and average cross-track errors with simulation results under the same current conditions. The results are shown in Table 8 and Table 9.

From Table 8 and Table 9, the maximum and average cross-track errors decrease with more trials, indicating that the proposed ILLT algorithm effectively learns in physical experiments. The addition of currents increases errors, as expected. Since simulations achieved good results by 30 iterations, and physical results are close to simulation values, the algorithm converged well under physical conditions. The slightly larger errors in experiments compared to simulations may be due to real-world random disturbances, delays, and sensor noise. Overall, the ILLT algorithm and transfer strategy are feasible and exhibit good reliability and robustness in physical experiments.

### 5.2. Seawater Tracking Experiment

**Figure 22:**
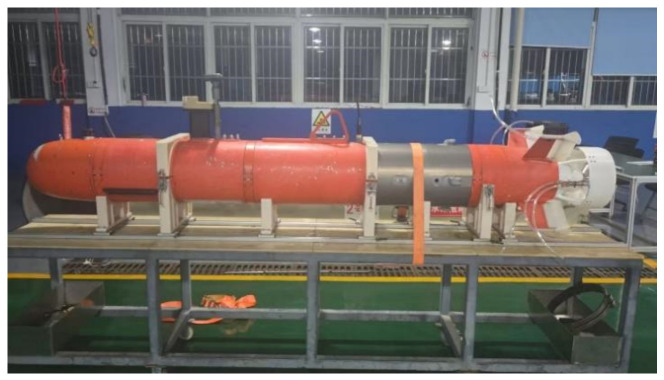
AUV used in the trials.

To verify the applicability of the algorithm in different environments, we conducted three-dimensional (3D) path-following experiments with the AUV in a seawater environment. In seawater, the current is uncontrollable, more random and general, and the underwater environment is more complex. The AUV mainly relies on its navigation system to accomplish path-following tasks. Since high-precision GPS positioning is ineffective underwater, a combined navigation system consisting of a Doppler velocity log (DVL) and a fiber-optic inertial navigation system (INS) was used for positioning in the experiments, while GPS was employed to calibrate the actual coordinates of the start and end points to eliminate the cumulative inertial errors of the combined navigation system. The AUV used in the trials is shown in Figure 22.

Because the combined positioning system only provides positioning in the *xoy* plane, and according to the earlier analysis, 3D path following can be decomposed into the *xoy* and *xoz* planes, we observed the tracking performance separately in both planes. The proposed algorithm was compared with traditional PID control. Consistent with the previous procedure, the AUV was first pre-trained in the simulation environment (source domain) and then tested in the actual seawater area using the proposed transfer strategy.

In the experiments, the start and end coordinates of the AUV motion were first determined, and a 3D predefined path was planned. The predefined path was decomposed into the *xoy* and *xoz* planes. In the *xoy* plane, the path consisted of straight-line segments connecting key points *P*_1_, *P*_2_, *P*_3_, and *P*_4_; similarly, in the *xoz* plane, the path consisted of straight-line segments connecting key points *Q*_1_, *Q*_2_, *Q*_3_, and *Q*_4_.

**Figure 23:**
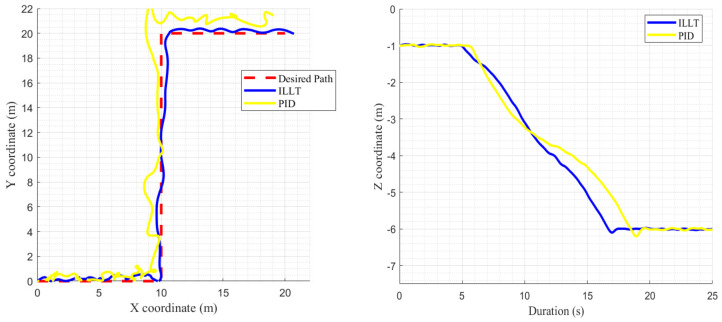
Trajectories in the xoy plane and depth variation under two control algorithms.

In the *xoy* plane, the positioning data recorded during AUV motion were used as the actual trajectory. In the *xoz* plane, the depth measurements from the depth sensor were combined with the positioning data: the depth values at the same time instants and the corresponding *x*-coordinates from the *xoy* plane were extracted to construct the actual motion curve in the *xoz* plane. The actual trajectories were compared with the predefined paths in both planes; a representative result is shown in Figure 23.

From Figure 23, it can be seen that the proposed ILLT algorithm effectively tracks the predefined path in the *xoy* plane, and achieves faster tracking and higher accuracy than PID control.

Plotting the depth z against the x-coordinate at the same time instants yields the actual AUV trajectories in the *xoz* plane under the different algorithms, as shown in Figure 23. It is evident that the ILLT algorithm also performs well in the *xoz* plane.

The experiment was repeated 10 times, and the key data were averaged for analysis, with the results summarized in Table 10. The values in parentheses are the source-domain simulation results.

As shown in Table 10, compared with PID control, the ILLT algorithm reduces the average cross-track error by 72.2% and 70.1% in the *xoy* and *xoz* planes, respectively, further confirming that the proposed ILLT algorithm can effectively solve the 3D path-following problem. The error values in the physical experiments are somewhat larger than those in simulations, mainly due to the uncertain currents in the actual environment and the errors of the positioning system. Under both control algorithms, the tracking error in the *xoz* plane is slightly larger than that in the *xoy* plane, primarily because the *xoz*-plane data are derived from different sensors. Overall, the proposed ILLT algorithm is effective and robust for 3D path-following in seawater environments.

## 6. Conclusions

This paper has addressed the path-following problem for underactuated AUVs under environmental disturbances by proposing a model-free deep reinforcement learning framework named ILLT (Improved LOS-LSTM-TD3). The algorithm integrates integral LOS guidance with the TD3 architecture, where the look-ahead distance is treated as a learnable optimization variable, and an LSTM network is introduced to capture temporal dependencies of AUV motion and path characteristics. A progressive unfreezing transfer strategy, combined with attention-based feature–current fusion and auxiliary consistency/regularization losses, enables efficient adaptation from simulation to target domains with varying currents.

Quantitative simulation results demonstrate that ILLT significantly outperforms the comparison algorithms. In the 2D source-domain experiments, the proposed method reduces the maximum and average cross-track errors by 47.8% and 48.5% compared to ILT (without LSTM), and by 50.4% and 66.4% compared to traditional PID control, respectively. In target-domain transfer under currents of *Vc* = [0.3,−0.1]*T* m/s, ILLT converges to a good policy within approximately 25 iterations, whereas the baseline without progressive unfreezing requires about 100 iterations. In 3D path-following experiments, ILLT achieves average cross-track errors of 0.23 m and 0.38 m in the xoy and xoz planes, respectively, representing reductions of 72.2% and 70.1% compared to PID. Physical experiments in both tank and lake environments further validate that the algorithm successfully transfers from simulation to reality, with cross-track errors in repeated trials approaching simulation values and success rates exceeding 90% under mild current conditions.

Despite these promising results, the current work has several limitations. First, the computational overhead of ILLT is higher than standard TD3 or PID due to the LSTM, dual critics, and attention modules, although a detailed runtime and memory benchmark on the embedded platform has not yet been conducted. Second, the policy is trained on specific path geometries (straight segments with sharp turns), and its generalization to significantly different shapes (e.g., sinusoidal or spiral paths) has not been tested. Third, the algorithm assumes that current velocity information is available; its robustness under noisy or delayed current estimates requires further investigation. Fourth, validation under stronger and time-varying currents has not been performed in the current revision cycle and remains an open question.

Future work will focus on the following directions: (1) systematic evaluation of ILLT under strong and dynamic ocean currents through extensive simulations; (2) quantitative profiling of inference time and memory usage on the embedded RK3588 platform to assess real-time deployability; (3) incorporation of online current estimation and uncertainty quantification to reduce reliance on accurate current measurements; (4) extension to more complex 3D paths and obstacle-aware path-following scenarios; and (5) exploration of lightweight network architectures to reduce computational cost for low-power embedded systems.

## Figures and Tables

**Table 1:** Design of the gradual unfreezing training strategy.

Trainable Modules	Training Progress p
Feature extractor fully frozen; only train current encoder, attention fusion, policy head, Q-heads	$0\leq p<\alpha_{1}$
Add unfreezing of LSTM layers in feature extractor	$\alpha_{1}\leq p<\alpha_{2}$
Add unfreezing of other layers	$\alpha_{2}\leq p<1$

**Table 2:** Schematic flowchart of the ILLT algorithm source-domain pre-training process.

Improved LOS-LSTM-TD3 (ILLT) Algorithm
Construct the underwater vehicle’s experience replay buffer D;
Set hyperparameters such as learning rate and discount factor;
Initialize Actor, two Critic networks, and their corresponding target networks;
For each episode do
Obtain the AUV’s initial environmental state s
Initialize LSTM hidden states
Initialize history window queue (fill with repeated first state or zeros)
For t=0 to step do
Select action $a_{t}$ based on current policy μ and state $s_{t}$ (with exploration noise added)
Execute the agent’s action, obtain reward $r_{t}$, and observe the new state $s_{t+1}$
Store the transition $\left(s_{t},a_{t},s_{t+1},r_{t}\right)$ in D
Sample a mini-batch of *N* transitions from D
Add smoothing noise ε to the target actions
Compute target value y according to Equation (25)
Update the Critic networks using gradient descent according to Equation (24)
if tmodd then
Update the Actor network using deterministic policy gradient according to Equation (26)
Update the target networks according to Equation (31)
end if
end for
end for

**Table 3:** Schematic flowchart of the ILLT algorithm target-domain transfer training process.

Improved LOS-LSTM-TD3 (ILLT) Algorithm
Load pre-trained model
Initialize gradual unfreezing scheduler, set unfreezing parameters
Initialize experience replay buffer D (for storing target-domain data)
for each episode do
Obtain the AUV’s initial environmental state s
For t=0 to step do
Select action $a_{t}$ based on current policy μ and state $s_{t}$ (with exploration noise added)
Execute the agent’s action, obtain reward $r_{t}$, and observe the new state $s_{t+1}$
Store the transition $\left(s_{t},a_{t},s_{t+1},r_{t}\right)$ in D
Check and execute gradual unfreezing: unfreeze modules, reinitialize optimizer
Sample a mini-batch of *N* transitions from D
Add smoothing noise ε to the target actions
Compute the total loss for the Critic networks according to Equation (29)
Update the Critic networks using gradient descent
if tmodd then
Add consistency loss and regularization loss
Update the Actor network using deterministic policy gradient according to Equation (30)
Update the target networks according to Equation (31)
end if
end for
end for

**Table 4:** Main parameter settings in the algorithm.

Parameter	Value
Soft update parameter τ	0.01
Discount factor γ	0.99
Learning rates for Actor and Critic networks	0.0002, 0.001
Replay buffer capacity	100,000
Loss function parameters $\lambda_{reg}$ and $\lambda_{cons}$	0.001, 0.05
Reward function parameters $\rho_{\varepsilon },\rho_{\psi },\rho_{\delta },\rho_{v},\rho_{\varepsilon }^{z},\rho_{\theta }$	2, 3, 1, 1, 2, 3
Penalty coefficients $k_{\varepsilon },k_{\psi },k_{\varepsilon }^{z},k_{\theta }$	6, 3, 6, 3

**Table 5:** Computed values of attention coefficient with varying current magnitudes.

Parameter	Value
$V_{c}={\left[0.1\;m/s,-0.1\;m/s\right]}^{T}$	0.12
$V_{c}={\left[0.3\;m/s,-0.1\;m/s\right]}^{T}$	0.25
$V_{c}={\left[0.5\;m/s,-0.4\;m/s\right]}^{T}$	0.47
$V_{c}={\left[0.8\;m/s,-0.8\;m/s\right]}^{T}$	0.66
$V_{c}={\left[1\;m/s,1\;m/s\right]}^{T}$	0.72
$V_{c}={\left[2\;m/s,2\;m/s\right]}^{T}$	0.91

**Table 6:** Comparison of key data during straight-line path tracking.

Control Method	ILLT Algorithm	ILT Algorithm	LLT Algorithm	Traditional PID
Max Cross-Track Error (m)	0.376	0.864	0.517	1.213
Avg Cross-Track Error (m)	0.121	0.293	0.178	0.450
Max Reward Value	−3.0	−7.4	−9.6	\
Reward Ratio at 100 Iterations	92.1	87.2	79.5	\

**Table 7:** Experimental camera parameters.

Parameter	Value
Focal length f/mm	3.15
Focal length in x-axis/mm	716.2
Focal length in y-axis/mm	714.8
Intrinsic matrix	$\left[\begin{array}{ccc} 716.2 & -15.9 & 777.6\\ 0 & 714.8 & 355.5\\ 0 & 0 & 1 \end{array}\right]$
Translation vector	$\left[\begin{array}{ccc} -107.5 & 2.5 & 6.8 \end{array}\right]$
Rotation matrix	$\left[\begin{array}{ccc} 0.999 & 0.006 & 0.009\\ -0.006 & 0.999 & 0.001\\ -0.009 & -0.001 & 0.999 \end{array}\right]$

**Table 8:** Maximum cross-track error (simulation values in parentheses).

Trial/Condition	1	10	20	30
$\left[v_{c}^{x},v_{c}^{y}\right]=\left[0,0\right]m/s$	0.7 (0.61)	0.67 (0.61)	0.66 (0.59)	0.63 (0.58)
$\left[v_{c}^{x},v_{c}^{y}\right]=\left[0.3,0\right]m/s$	1.2	0.85	0.77	0.71 (0.69)
$\left[v_{c}^{x},v_{c}^{y}\right]=\left[0,0.4\right]m/s$	1.28	0.9	0.79	0.73 (0.72)

**Table 9:** Average cross-track error (simulation values in parentheses).

Trial/Condition	1	10	20	30
$\left[v_{c}^{x},v_{c}^{y}\right]=\left[0,0\right]m/s$	0.2 (0.16)	0.18 (0.16)	0.17 (0.15)	0.16 (0.15)
$\left[v_{c}^{x},v_{c}^{y}\right]=\left[0.3,0\right]m/s$	0.6	0.42	0.25	0.21 (0.19)
$\left[v_{c}^{x},v_{c}^{y}\right]=\left[0,0.4\right]m/s$	0.7	0.47	0.28	0.22 (0.21)

**Table 10:** Key data comparison for path tracking.

Control Method	Average Cross-Track Error	
	xoy Plane	xoz Plane
PID	0.83	1.27
ILLT	0.23 (0.15)	0.38 (0.16)

## Data Availability

The data presented in this study are not publicly available due to privacy restrictions. They are available on request from the corresponding author.
